# Glucose monitoring in living cells with single fluorescent protein-based sensors†

**DOI:** 10.1039/c7ra11347a

**Published:** 2018-01-10

**Authors:** Hanyang Hu, Yufeng Wei, Daocheng Wang, Ni Su, Xianjun Chen, Yuzheng Zhao, Guixia Liu, Yi Yang

**Affiliations:** Synthetic Biology and Biotechnology Laboratory, State Key Laboratory of Bioreactor Engineering, Shanghai Collaborative Innovation Center for Biomanufacturing Technology, East China University of Science and Technology 130 Mei Long Road Shanghai 200237 China; Optogenetics & Synthetic Biology Interdisciplinary Research Center, CAS Center for Excellence in Brain Science, Shanghai Institutes for Biological Sciences, Chinese Academy of Sciences Shanghai 200031 China yiyang@ecust.edu.cn; Shanghai Key Laboratory of New Drug Design, School of Pharmacy, East China University of Science and Technology 130 Mei Long Road Shanghai 200237 China gxliu@ecust.edu.cn

## Abstract

Glucose is the main source of energy and carbon in organisms and plays a central role in metabolism and cellular homeostasis. However, the sensitive fluctuation of glucose in living cells is difficult to monitor. Thus, we developed a series of ratiometric, highly responsive, single fluorescent protein-based glucose sensors of wide dynamic range by combining a circularly permuted yellow fluorescent protein with a bacterial periplasmic glucose/galactose-binding protein. We used these sensors to monitor glucose transport in living *Escherichia coli* cells, and found that the cells take up glucose within 10 min to maintain physiological glucose levels, and observed the differences in glucose uptake and glucose metabolism between wild-type and Mlc knockout cells. These sensors can be specific and simple tools for glucose detection *in vitro* and non-invasive tools for real-time monitoring of glucose metabolism *in vivo*.

## Abstract 2

Glucose is the main source of energy and carbon in organisms and plays a central role in metabolism and cellular homeostasis.

## Graphical abstract

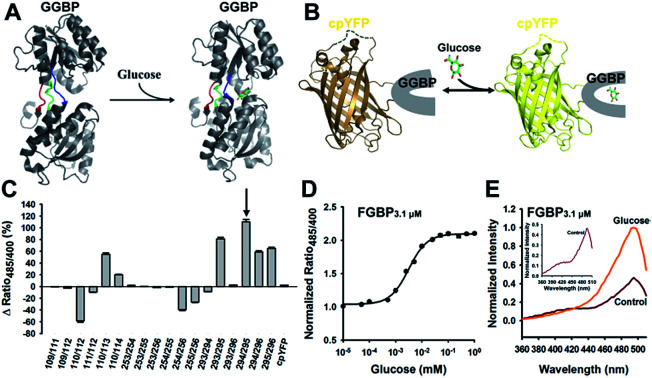

## 1. Introduction

Glucose is the main source of energy and carbon in most organisms, from bacteria to humans. Changes in glucose uptake, release, and metabolism are associated with regulation of various physiological and pathological phenomena, such as cell growth, cell proliferation, cell differentiation, cell death, insulin secretion, obesity, and diabetes.^1–3^ Therefore, sensitive and selective measurement of glucose has become significant. Radiolabeling, chromatography, and mass spectrometry have been used effectively to quantify glucose.^4^ However, these methods have limited spatiotemporal resolution and are unsuitable for real-time imaging of glucose metabolism in living cells.

To monitor glucose *in situ*, fluorescence resonance energy transfer (FRET)-based glucose biosensors with different affinity have been developed and proven to be useful for imaging glucose flux in living cells.^5–10^ These sensors are based on enhanced cyan fluorescent protein/enhanced yellow fluorescent protein FRET pair and glucose/galactose-binding protein (GGBP) of *Escherichia coli*. GGBP is a bacterial periplasmic-binding protein that exhibits a hinge-twist conformational change upon glucose binding.^11–13^ Unfortunately, the fluorescence changes of these FRET sensors are small, which fall into 10–70%,^5–10^ and this limits their applications in high-throughput screen and monitoring subtle metabolic changes.

Recently, we have reported a series of metabolite sensors, including NADH (Frex),^14^ NAD+/NADH ratio (SoNar),^15,16^ NADPH (iNap)^17^ and histidine (FHisJ)^18^ based on circularly permuted yellow fluorescent protein (cpYFP). In cpYFP, the original N- and C-termini are fused by a polypeptide linker, and new termini are introduced close to the fluorophore, making its fluorescence highly sensitive to the protein's conformation.^14–18^ Compared with FRET-based sensors, cpYFP-based sensors often have larger fluorescence changes; in specific, Frex,^14^ SoNar,^15,16^ iNap^17^ and FHisJ^18^ sensors have more than 500% dynamic range, rendering them capable of tracking subtle metabolic changes.

To maximize the existing technical advantages, we inserted cpYFP into three linker locations of GGBP and developed a series of ratiometric, highly responsive, single fluorescent protein-based glucose sensors, denoted as FGBP (fluorescent GGBP). Among them, FGBP_1 mM_ sensor with a binding constant (*K*_d_) of 1.0 mM exhibited sevenfold fluorescence ratio changes and fit physiological applications.

## 2. Experimental

### 2.1 Plasmid construction and strains

To generate single fluorescent protein-based glucose indicators, the MglB DNA sequence coding for the mature GGBP (positions 70–927 relative to ATG) was amplified from *E. coli* genomic DNA by PCR with the primers P1 and P2 (sequences available in Table S1†) and cloned into *BamHI-HindIII* sites in the pRSETb vector (Invitrogen). The GGBP–cpYFP insertion variants were constructed by overlap PCR^19^ using wild-type GGBP sequence and cpYFP from Frex.^14^ First, the coding sequences of N and C terminal domain of GGBP, and cpYFP were amplified using the primers P1 and PR, PF and P2, and P3 and P4, respectively (sequences available in Table S1†). Second, the chimeric construct consisting of GGBP and cpYFP was produced using an overlapping PCR with the primers P1 and P2. This product was cloned into the BamHI/HindIII sites of pRSETb (Invitrogen), yielding pRSETb-FGBP (GGBP and cpYFP chimeras) (Fig. S1A and S1B†). We truncated the N and C terminal amino acid residue of cpYFP to expand the dynamic range of FGBP by PCR (Fig. S1C†). To improve the affinity of the sensors, sited directed mutagenesis of FGBP_27 μM_ was generated by PCR (Fig. S1D†). The electrophoresis data showed the protein size of different sensors (Fig. S1E†).

Mlc knockout strain is a derivative of *E. coli* JM109 (DE3). Mlc was deleted according to the method of Datsenko and Wanner^20^ using plasmid pKD4 or pKD3, leaving the start codon and seven codons at the 3′ end of the target gene. The resistance cassettes were eliminated as described previously.^20^ To overexpress Mlc, the Mlc gene was amplified from *E. coli* genomic DNA by PCR and cloned into *BamHI-HindIII* sites in the pCDFDuet1 vector (Novagen).

### 2.2 Library construction

Preliminary truncations of the N- and C-terminals of cpYFP indicated that deletions beyond five AAs of the N- and C-terminals (data not shown) caused misfoldings. Therefore, the library was limited to the deletions of four AAs of the N- and C-terminals. Truncation combinations were amplified with Primers STAR HS DNA polymerase (Takara) and fused by T4 DNA ligase (Fermentas). Mutants with different affinities were engineered in FGBP_27 μM_, using overlap PCR,^19^ and then transferred to the pRSETb vector. The DNA sequence and amino acid sequence of FGBP_1 mM_ are in the “ESI note†” section.

### 2.3 Protein expression and *in vitro* characterization

All recombinant proteins with a His_6_-tag were expressed in *E. coli* JM109 (DE3) by the pRSETb expression plasmid as previously described.^18^ Proteins were purified with Ni–NTA His SpinTrap column.

Spectral measurement was performed in 20 mM MOPS buffer (pH 7.4) by using a spectrofluorometer (EnSpire). Excitation spectra were recorded between 350 and 510 nm, and emission at 528 nm as previously described.^14,15^

For glucose titration, the protein was diluted in 20 mM MOPS buffer (pH 7.4) to a final concentration of 1 μM. The fluorescence value of protein was measured by a filter-based Synergy 2 Multi-Mode microplate reader using 400 BP 10 nm, 485 BP 20 nm excitation, and 528 BP 20 nm emission (BioTek). The ratio (*R*) was defined as the fluorescence intensity at 485 nm divided by the intensity at 400 nm.

The *K*_d_ of each glucose sensor was determined by fitting to a single-site binding isotherm:1
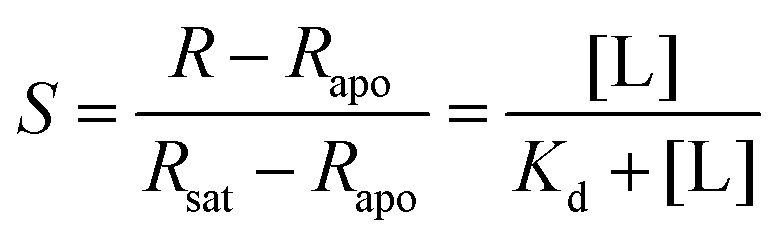
where *S* is the fraction of sensor saturation, [L] is the concentration of glucose, *R* is the fluorescence ratio_485/400_ of sample, *R*_apo_ is the ratio in the absence of ligand, and *R*_sat_ is the ratio at saturation with ligand.

### 2.4 Monitoring glucose transport in *E. coli* cells

To monitor glucose transport in living cells, *E. coli* JM109 (DE3) cells carrying pRSETb-FGBP_1 mM_ were grown in Luria-Bertani medium containing 100 μg ml^−1^ ampicillin at 37 °C until the cultures reached about 0.6–0.8 OD. The expression of proteins was induced by 0.1 mM IPTG at 18 °C overnight. *E. coli* cells were harvested by centrifugation at 4000 × *g* for 30 min at 4 °C, washed twice with M9 mini medium (pH 7.4, containing 100 mM HEPES), and then incubated in the same buffer for 6 h to ensure low endogenous glucose levels.

After 6 h starving, *E. coli* cells were harvested by centrifugation, washed twice with M9 mini medium (pH 7.4, containing 100 mM HEPES), and then suspended in the same buffer. Subsequently, 45 μl cultures (0.5 OD) were transferred into 384 well plates. Fluorescence emission at 528 nm (excitation at 400 and 485 nm) was recorded for 5 min by Synergy 2 Multi-Mode microplate reader. M9 mini medium (5 μl) with different glucose concentrations was added manually to the cultures. Fluorescence was then measured for 30 min. Fluorescence values were background corrected by subtracting the intensity of *E. coli* JM109 (DE3) cell samples not expressing sensors.

## 3. Results and discussion

### 3.1 Generation of cpYFP-based sensors for glucose

**Fig. 1:**
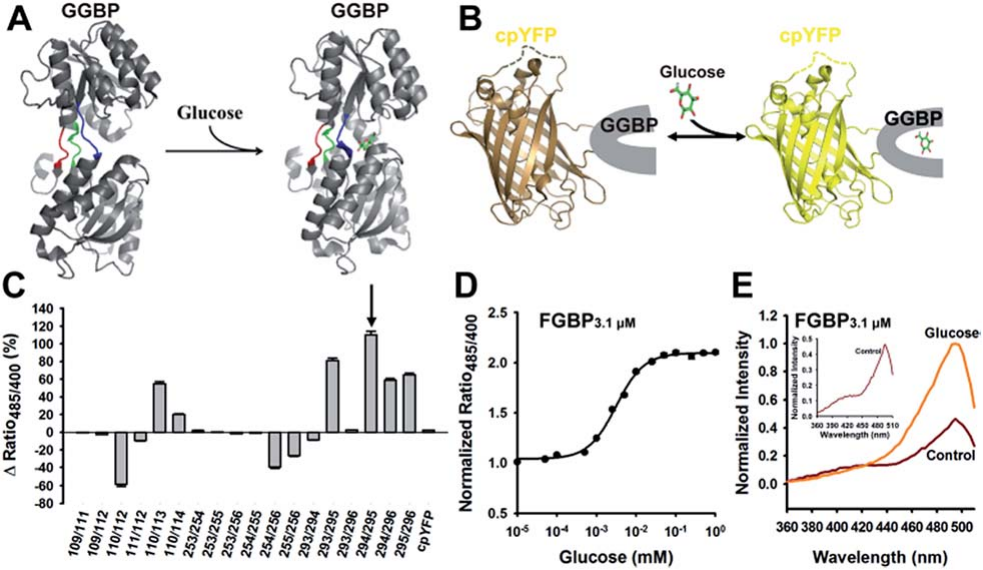
Generation of cpYFP-based glucose sensors. (A) Crystallographic structures of glucose-free (PDB ID code 2FW0) and glucose-binding (PDB ID code 2FVY) GGBP from *E. coli* are drawn using the molecular-graphics software PyMOL based on Protein Data Bank files. The colored ribbon parts [residues 109–114 (blue), 253–256 (green), 293–296 (red)] represent the flexible and target region for the insertion of cpYFP to generate glucose indicators. (B) Design of glucose sensor, in which cpYFP was inserted into the flexible linker region of GGBP. Binding of glucose (green and red) changes protein conformation and fluorescence. (C) Comparison of cpYFP and 18 sensor variants in glucose responsiveness. (D) Titration of the chimeric Pro294/Tyr295 protein named as FGBP_3.1 μM_. Fluorescence ratios were normalized to the control condition in the absence of glucose. (E) Excitation spectra of FGBP_3.1 μM_ with or without 100 mM glucose, normalized to the peak intensity in the glucose condition. Emission was measured at 528 nm. For C and D, data are presented in three biological replicates, and error bars represent SEM.

To engineer a cpYFP-based indicator for glucose, bacterial periplasmic GGBP^11–13^ was chosen as a suitable glucose-binding detector because of the following findings: (1) glucose binds to GGBP with high specificity^11,12^ and (2) glucose binding to GGBP results in a dramatic conformational change demonstrated by X-ray and NMR analyses,^13^ as shown in Fig. 1A. In addition, GGBP was used as a sensor domain of FRET-based glucose indicators as previously reported.^8,9^

According to crystallographic structures of GGBP,^13,18^ chimeric proteins were generated by inserting cpYFP into the three flexible linker regions of GGBP, namely, Gly109–Glu114, Thr253–Asn256, and Val293–Val296 (Fig. 1A and B). Among them, the chimera with cpYFP inserted between Pro294 and Tyr295 of GGBP showed a 2.1-fold increase in the ratio of fluorescence when excited at 485 and 400 nm upon glucose addition (Fig. 1C, Table 1). Fluorescence titration studies showed that the Pro294/Tyr295 chimera named FGBP_3.1 μM_ had an apparent dissociation constant (*K*_d_) ∼3.1 μM for glucose at pH 7.4 (Fig. 1D, Table 1). Similar to other cpYFP-based sensors,^17,18^ FGBP_3.1 μM_ has two typical excitation peaks around 420 and 500 nm and one emission peak near 515 nm (Fig. 1E).

**Table 1:** Properties of five glucose sensors^a^^,^^b^

Sensor	Engineering	*R*_sat_/*R*_apo_ (%)	*K*_d_ (mM)	Detection range (mM)
FGBP_3.1 μM_	P294/Y295	210	0.0031	0.0005–0.02
FGBP_27 μM_	P294/Y295, N3C4	590	0.027	0.0009–0.86
FGBP_380 μM_	P294/Y295, N3C4, N256S	510	0.38	0.014–10
FGBP_1 mM_	P294/Y295, N3C4, L238S	690	1.0	0.026–38
FGBP_3.2 mM_	P294/Y295, N3C4, A213R	630	3.2	0.09–110

a P294/Y295, cpYFP was inserted between Pro294 and Tyr295 amino acids of GGBP.

b N3C4, N-terminal 3 amino acids and C-terminal 4 amino acids of cpYFP were truncated.

### 3.2 Optimization improves the responsiveness and affinity of FGBP sensors

**Fig. 2:**
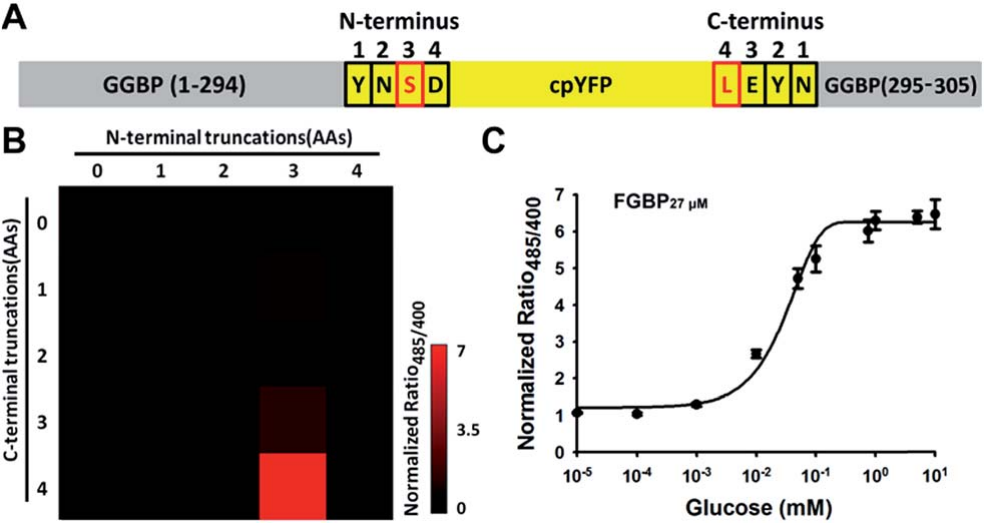
Optimization improves the responsiveness of glucose sensor. (A and B) Truncation mutants of Pro294/Tyr295 (FGBP_3.1 μM_) (A) and their fluorescence response (B) toward 100 mM glucose. The most responsiveness mutant N3C4 named FGBP_27 μM_ is indicated in red boxes. (C) Titration curve of FGBP_27 μM_. Fluorescence ratios were normalized to the control condition in the absence of glucose. Data are presented in three biological replicates, and error bars represent SEM.

To maximize the response magnitude of sensors, we created a cpYFP-terminal truncation library between GGBP and cpYFP of FGBP_3.1 μM_ (Fig. 2A) and found that the FGBP_3.1 μM_ variant N3C4 manifests the most dramatic increase in the presence of glucose, as measured by the ratio of fluorescence excited at 485 and 420 nm (Fig. 2B). Fluorescence titration studies showed that the N3C4 variant (named FGBP_27 μM_) had an apparent *K*_d_ of ∼27 μM for glucose (Fig. 2C and Table 1).

**Fig. 3:**
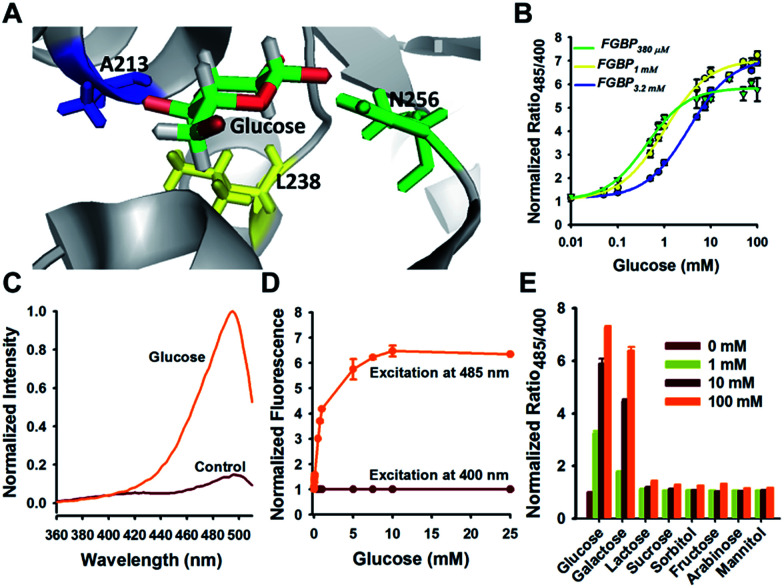
Optimization tunes the affinity of glucose sensor. (A) Crystallographic structure of GGBP (PDB ID code 2FVY) showing the glucose-binding pocket. Three sites (A213, L238, and N256) are potential for sensor affinity improvement. (B) Titration curves of A213R (named FGBP_3.2 mM_), L238S (named FGBP_1 mM_), and N256S (named FGBP_380 μM_) mutants. (C) Excitation spectra of FGBP_1 mM_ with or without 100 mM glucose, normalized to the peak intensity in the glucose condition. Emission was measured at 528 nm. (D) Fluorescence intensities of FGBP_1 mM_ with excitation at 400 or 485 nm normalized to the initial value; emission at 528 nm. (E) Substrate specificity of FGBP_1 mM_ as measured by ratio_485/400_ in the presence of various sugar ligands. Data were normalized to the control condition in the absence of ligand. For B, D, and E, data are presented in three biological replicates, and error bars represent SEM.

Physiological glucose level has been estimated in the range of 0.4–24 mM, such as 2–5 mM in plants,^7^ 1.5 mM in yeast,^21^ 3–9 mM in blood,^22^ 1–10 mM in liver,^23^ and 0.4–24 mM in the intestine,^24^ far exceeding the dissociation constants of FGBP_27 μM_ for glucose. To tune the affinity of the FGBP_27 μM_ sensor, we further created variants of the sensor with single site-directed mutagenesis of three key amino acid residues around the glucose binding pocket (Fig. 3A).^25–27^ The three mutants, *i.e.*, N256S, L238S, and A213R, had different affinities, with apparent *K*_d_ values of ∼380 μM, ∼1.0 mM, and ∼3.2 mM, and were denoted as FGBP_380 μM_, FGBP_1 mM_, and FGBP_3.2 mM_, respectively (Fig. 3B and Table 1). Considering the maximum fluorescence ratio change, affinity, and expression level, we chose FGBP_1 mM_, which covers the physiological blood glucose range, for further characterization (Table 1).

Similar to FGBP_3.1 μM_, FGBP_1 mM_ also has two typical excitation peaks around 420 and 500 nm and one emission peak near 515 nm (Fig. 3C). Upon glucose binding, the fluorescence of FGBP_1 mM_ excited at 485 nm showed a 6.5-fold increase, and the fluorescence excited at 400 nm was almost constant (Fig. 3D). Apart from glucose and galactose, none of the other sugars tested induced a significant change in ratio at 1 and 100 mM concentrations (Fig. 3E), showing the high selectivity of FGBP_1 mM_ toward glucose. Glucose is expected to be present in significantly higher concentrations than galactose; thus, FGBP_1 mM_ is suitable for glucose monitoring in living cells.

**Fig. 4:**
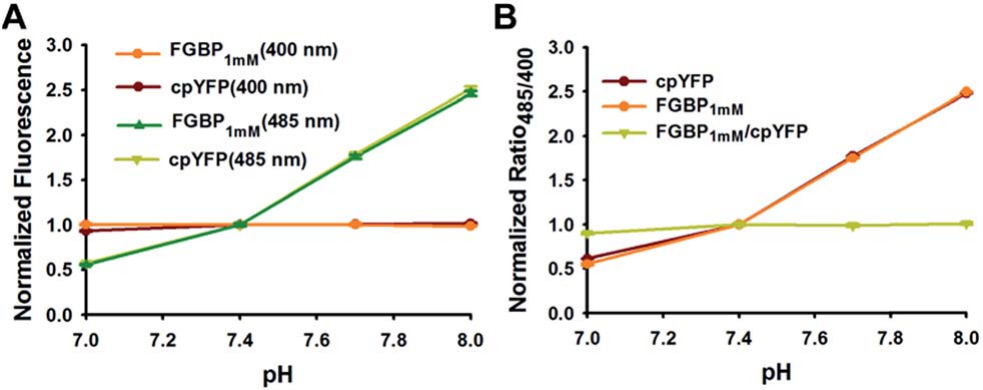
Effect of pH on FGBP1 mM and cpYFP. (A) Fluorescence intensities of FGBP1 mM and cpYFP with excitation at 400 nm or 485 nm, and emission at 528 nm. (B) pH-dependency of the excitation ratio _485/400_ of FGBP_1 mM_ and cpYFP. For A and B, data are normalized to the fluorescence or ratio at pH 7.4, and are presented in three biological replicates, and error bars represent SEM.

Similar to all other genetically encoded sensors based on cpYFP, FGBP_1 mM_ depended on pH when excited at 485 nm; however, FGBP_1 mM_ fluorescence excited at 400 nm is much more pH resistant (Fig. 4A). At modest pH fluctuations, the pH effects of FGBP_1 mM_ can be corrected by measuring the fluorescence of FGBP_1 mM_ and cpYFP in parallel, due to their very similar pH responses (Fig. 4B).

### 3.3 Real-time monitoring of intracellular glucose in living cells

**Fig. 5:**
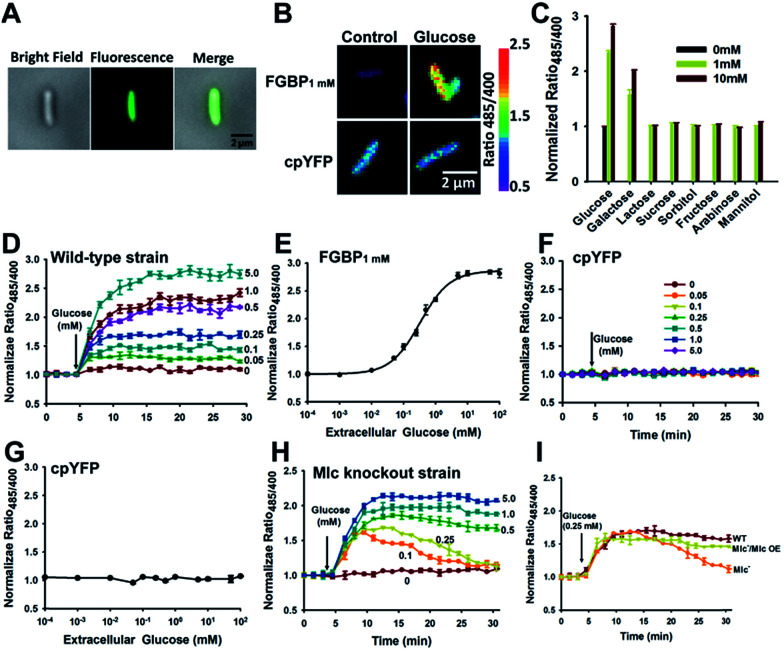
Real-time monitoring of intracellular glucose in living cells. (A) Fluorescence images of FGBP_1 mM_-expressing *E. coli* JM109 (DE3) cells. (B) Fluorescence images of FGBP_1 mM_ or cpYFP-expressing cells before and after incubation with 25 mM glucose. (C) Fluorescence responses of FGBP_1 mM_ in live cells treated with exogenous glucose and its analogs. (D and F) Kinetics of FGBP_1 mM_ (D) or cpYFP (F) fluorescence responses in *E. coli* JM109 (DE3) cells treated with exogenous glucose. (E and G) Fluorescence responses of FGBP_1 mM_ (E) or cpYFP (G) in *E. coli* JM109 (DE3) cells after glucose addition for 20 min. (H) Kinetics of FGBP_1 mM_ fluorescence responses in Mlc knockout *E. coli* JM109 (DE3) cells treated with exogenous glucose. (I) Kinetics of FGBP_1 mM_ fluorescence responses to glucose in wild-type or Mlc knockout *E. coli* JM109 (DE3) cells. For C–I, data are presented in three biological replicates, and error bars represent SEM.

The ability of transport glucose across the plasma membrane is a common feature to nearly all cells, from the simple bacterium to the highly compartmented mammalian cells.^28^ To test the ability of FGBP_1 mM_ to report changes in intracellular glucose levels, we expressed the FGBP_1 mM_ sensor in living *E. coli* JM109 (DE3) cells. Fluorescence was uniform throughout the cell, suggesting that this sensor was located in the cytoplasm but not cell surface (Fig. 5A). Addition of exogenous glucose into the culture medium induced a rapid, dose-dependent, and saturable increase in the fluorescence ratio (Fig. 5B–E), whereas addition of glucose analogs had no effect on fluorescence (Fig. 5C). This finding suggested that glucose was readily transported across the cell membrane of these bacteria. Michaelis–Menten fitting of FGBP_1 mM_'s fluorescence ratio *versus* extracellular glucose concentration produced a *K*_0.5_ of ∼0.3 mM (Fig. 5E), which is much higher than the *K*_m_ for ptsG in *E. coli*, the high-affinity glucose transporter in the plasma membrane.^29^ By contrast, only slight changes in the fluorescence ratio were observed in *E. coli* JM109 (DE3) cells expressing cpYFP instead of FGBP_1 mM_ when glucose was added to the cell culture medium (Fig. 5F and G), excluding the possibilities of fluorescence interference of pH variations of the cpYFP domain.

The phosphotransferase system (PTS) is the major sugar transport system in many Gram-positive and Gram-negative bacterial species; however, expression of ptsG is repressed by the Mlc (making large colonies) protein.^30^ To investigate the role of Mlc on glucose transport, we constructed Mlc knockout *E. coli* JM109 (DE3) cells. Compared with wild-type cells, we surprisingly found that glucose-induced the increase of fluorescence gradually returned to basal levels as the extracellular glucose was consumed in Mlc knockout cells (Fig. 5D and H), and Mlc overexpression rendered the similarity in metabolic kinetics of these cells (Fig. 5I). These results imply that Mlc expression level not only regulates glucose uptake but also influences the rate of glucose metabolism.

## 4. Conclusions

In this work, we reported a series of ratiometric, highly specific, highly sensitive, and single fluorescent protein-based glucose sensors with different affinities. Among them, FGBP_1 mM_ can detect glucose in the range of 0.02–40 mM, which covers the physiological glucose concentration in organisms. FGBP_1 mM_ displays a large dynamic range and is very useful for the real-time tracking of subtle changes in cell metabolism. FGBP_1 mM_ displays a ∼700% fluorescence change *in vitro*, almost 10-fold greater than that of previously reported FRET-based glucose sensors,^5–10^ rendering it a highly responsive genetic-encoded sensor. Compared with FRET-based glucose sensors, FGBP sensors only have one fluorescent protein and are intrinsically ratiometric, allowing the built-in normalization of the fluorescence signals irrespective of variations in indicator protein concentrations. Considering the admirable properties of these sensors, we believe that FGBP sensors could be good alternatives to existing methods for intracellular glucose detection.

## Conflicts of interest

The authors have declared no conflicts of interest.

## Supplementary Material

RA-008-C7RA11347A-s001
