# Supplementary material for: Glucose monitoring in living cells with single fluorescent protein-based sensors
Source: RSC Adv. 2018 Jan 10;8(5):2485–9. doi: 10.1039/c7ra11347a (PMC9077360; doi:10.1039/c7ra11347a)
Supplement: RA-008-C7RA11347A-s001 [file RA-008-C7RA11347A-s001.pdf]

Electronic Supplementary Material (ESI) for RSC Advances.
This journal is © The Royal Society of Chemistry 2018

Electronic Supplementary Information

Glucose monitoring in living cells with single fluorescent protein-based sensors

Hanyang Hu,[ac] Yufeng Wei,[ac] Daocheng Wang, [ac] Ni Su,[ac] Xianjun Chen, [ac] Yuzheng Zhao, [ac] Guixia Liu*[c] and Yi Yang* [ab]

a. Synthetic Biology and Biotechnology Laboratory, State Key Laboratory of Bioreactor Engineering, Shanghai Collaborative Innovation Center for Biomanufacturing Technology, , East China University of Science and Technology, 130 Mei Long Road, Shanghai 200237, China

b. Optogenetics & Synthetic Biology Interdisciplinary Research Center, CAS Center for Excellence in Brain Science, Shanghai Institutes for Biological Sciences, Chinese Academy of Sciences, Shanghai 200031, China. E-mail: yiyang@ecust.edu.cn

c. Shanghai Key Laboratory of New Drug Design, School of Pharmacy, East China University of Science and Technology, 130 Mei Long Road, Shanghai 200237, China. E-mail: gxliu@ecust.edu.cn

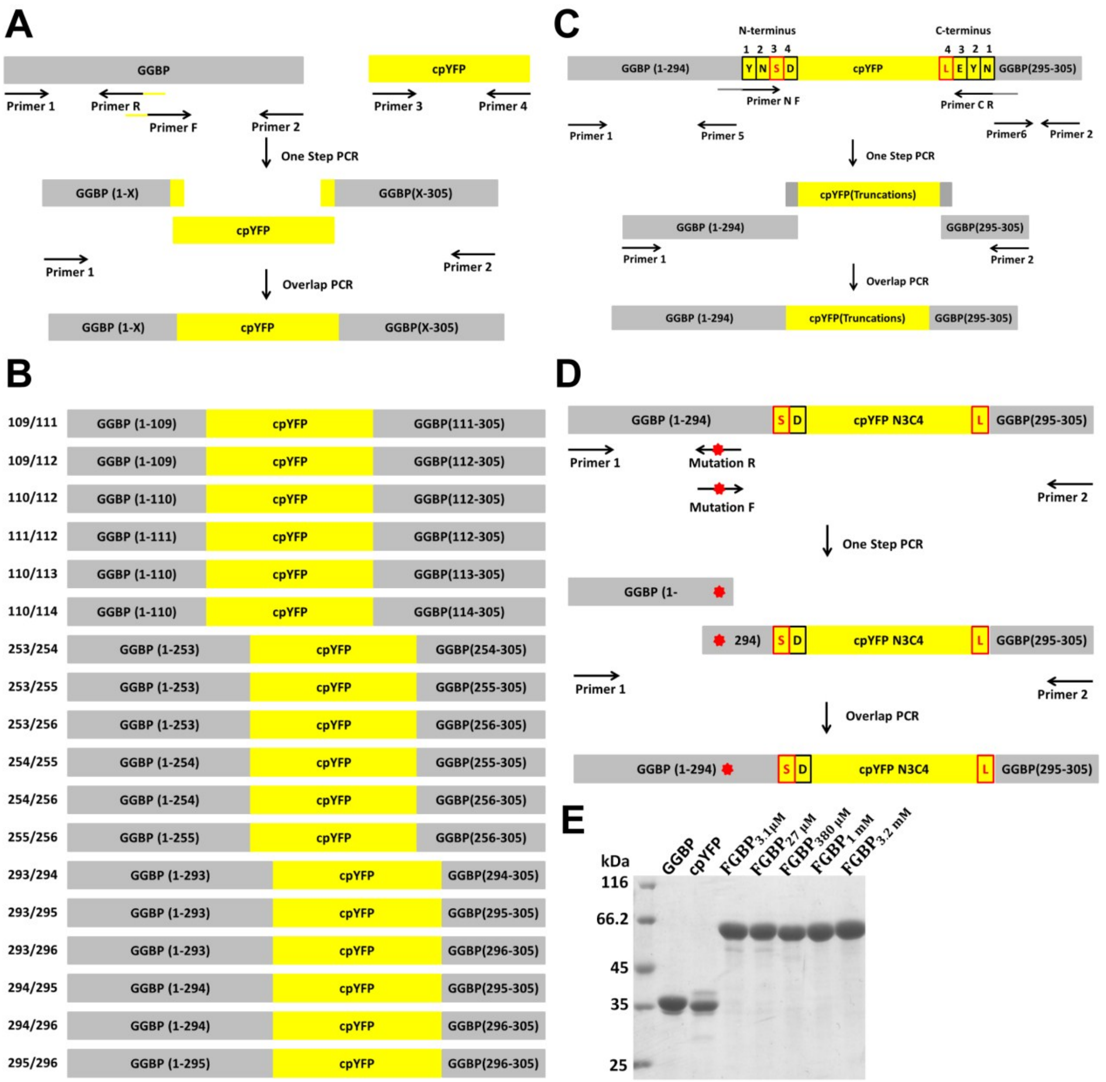

Fig. S1 (A) GGBP and cpYFP chimeras were obtained by overlap PCR. (B) Schematic model for eighteen GGBP and cpYFP chimeras. (C) GGBP and truncated cpYFP chimeras were obtained by overlap PCR. (D) $FGBP_{27\ \mu M}$ variants were obtained by overlap PCR. (E) Purified sensor proteins were resolved with 10% SDS-PAGE.

Table S1 Primers Used for FGBP Construction, Truncation, and Mutation

Primer	Sequence 5` to 3`
1	CCCGGATCCGGCTGATACTCGCATTGGT
2	CCCAAGCTTTTATTTCTTGCTGAATTCAGC
3	TACAACAGCGACAACGTC
4	GTTGTACTCCAGCTTGTG
109 R	GACGTTGTCGCTGTTGTAGCCAACGTAGTAGGCTTT
110 R	GACGTTGTCGCTGTTGTAAGTGCCAACGTAGTAGGC
111 R	GACGTTGTCGCTGTTGTAGTCAGTGCCAACGTAGTA
111 F	CACAAGCTGGAGTACAACGACTCCAAAGAGTCCGGC
112 F	CACAAGCTGGAGTACAACTCCAAAGAGTCCGGCATT
113 F	CACAAGCTGGAGTACAACAAAGAGTCCGGCATTATT
114 F	CACAAGCTGGAGTACAACGAGTCCGGCATTATTCAA
253 R	GACGTTGTCGCTGTTGTAGGTGCCCGCCAGTGCACC
254 R	GACGTTGTCGCTGTTGTATACGGTGCCCGCCAGTGC
255 R	GACGTTGTCGCTGTTGTACAGTACGGTGCCCGCCAG
254 F	CACAAGCTGGAGTACAACGTACTGAACGATGCTAAC
255 F	CACAAGCTGGAGTACAACCTGAACGATGCTAACAAC
256 F	CACAAGCTGGAGTACAACAACGATGCTAACAACCA
293 R	GACGTTGTCGCTGTTGTATACGCGGACCACTTTGTT
294 R	GACGTTGTCGCTGTTGTAAGGTACGCGGACCACTTT
295 R	GACGTTGTCGCTGTTGTAATAAGGTACGCGGACCAC
294 F	CACAAGCTGGAGTACAACCCTTATGTTGGCGTAGAT
295 F	CACAAGCTGGAGTACAACTATGTTGGCGTAGATAAA
296 F	CACAAGCTGGAGTACAACGTTGGCGTAGATAAAGAC
5	AGGTACGCGGACCACTTT
6	TATGTTGGCGTAGATAAA
cpYFP N1 F	AAAGTGGTCCGCGTACCTAACAGCGACAACGTCTAT
cpYFP N2 F	AAAGTGGTCCGCGTACCTAGCGACAACGTCTATATC
cpYFP N3 F	AAAGTGGTCCGCGTACCTGACAACGTCTATATCATG
cpYFP N4 F	AAAGTGGTCCGCGTACCTAACGTCTATATCATGGCC
cpYFP C1 R	TTTATCTACGCCAACATAGTACTCCAGCTTGTGCCC
cpYFP C2 R	TTTATCTACGCCAACATACTCCAGCTTGTGCCCCAG
cpYFP C3 R	TTTATCTACGCCAACATACAGCTTGTGCCCCAGGAT
cpYFP C4 R	TTTATCTACGCCAACATACTTGTGCCCCAGGATGTT
A213R F	CGCCAACAACGATCGTATGGCAAT
A213R R	ATTGCCATACGATCGTTGTTGGCG
L238S F	TGGCGTCGATGCGTCTCCAGAAGC
L238S R	GCTTCTGGAGACGCATCGACGCCA
N256S F	GGGCACCGTACTGTCTGATGCTAA
N256S R	TTAGCATCAGACAGTACGGTGCCC

Supplementary Note

The DNA sequence of $FGBP_{1\ mM}$

ATGGCTGATACTCGCATTGGTGTAACAATCTATAAGTATGATGATAACTTTATGTCTGTAGTGCGCAAGGC
TATTGAGCAAGATGCGAAAGCCGCGCCAGATGTTCAGCTGCTGATGAATGATTCTCAGAATGACCAGTCC
AAGCAGAACGATCAGATCGACGTATTGCTGGCGAAAGGGGTGAAGGCACTGGCAATCAACCTGGTTGAC
CCGGCAGCTGCGGGTACGGTGATTGAGAAAGCGCGTGGGCAAAACGTGCCGGTGGTTTTCTTCAACAAA
GAACCGTCTCGTAAGGCGCTGGATAGCTACGACAAAGCCTACTACGTTGGCACTGACTCCAAAGAGTCCG
GCATTATTCAAGGCGATTTGATTGCTAAACACTGGGCGGCGAATCAGGGTTGGGATCTGAACAAAGACG
GTCAGATTCAGTTCGTACTGCTGAAAGGTGAACCGGGCCATCCGGATGCAGAAGCACGTACCACTTACGT
GATTAAAGAATTGAACGATAAAGGCATCAAAACTGAACAGTTACAGTTAGATACCGCAATGTGGGACACC
GCTCAGGCGAAAGATAAGATGGACGCCTGGCTGTCTGGCCCGAACGCCAACAAAATCGAAGTGGTTATC
GCCAACAACGATGCGATGGCAATGGGCGCGGTTGAAGCGCTGAAAGCACACAACAAGTCCAGCATTCCG
GTGTTTGGCGTCGATGCGTCTCCAGAAGCGCTGGCGCTGGTGAAATCCGGTGCACTGGCGGGCACCGTA
CTGAACGATGCTAACAACCAGGCGAAAGCGACCTTTGATCTGGCGAAAAACCTGGCCGATGGTAAAGGT
GCGGCTGATGGCACCAACTGGAAAATCGACAACAAAGTGGTCCGCGTACCTGACAACGTCTATATCATGG
CCGACAAGCAGAAGAACGGCATCAAGGCCTACTTCAAGATCCGCCACAACGTCGAGGACGGCAGCGTGC
AGCTCGCCGACCACTACCAGCAGAACACCCCCATCGGCGACGGCCCCGTGCTGCTGCCCGACAACCACTA
CCTGAGCTTCCAGTCCGTCCTGAGCAAAGACCCCAACGAGAAGCGCGATCACATGGTCCTGCTGGAGTTC
GTGACCGCCGCCGGGATCACTCTCGGCATGGACGAGCTGTACAACGTGGATGGCGGTAGCGGTGGCACC
GGCAGCAAGGGCGAGGAGCTGTTCACCGGGGTGGTGCCCATCCTGGTCGAGCTGGACGGCGACGTAAA
CGGCCACAAGTTCAGCGTGTCCGGCGAGGGCGAGGGCGATGCCACCTACGGCAAGCTGACCCTGAAGCT
GATCTGCACCACCGGCAAGCTGCCCGTGCCCTGGCCCACCCTCGTGACCACCCTCGGCTACGGCCTGAAG
TGCTTCGCCCGCTACCCCGACCACATGAAGCAGCACGACTTCTTCAAGTCCGCCATGCCCGAAGGCTACGT
CCAGGAGCGCACCATCTTCTTCAAGGACGACGGCAACTACAAGACCCGCGCCGAGGTGAAGTTCGAGGG
CGACACCCTGGTGAACCGCATCGAGCTGAAGGGCATCGGCTTCAAGGAGGACGGCAACATCCTGGGGCA
CAAGTATGTTGGCGTAGATAAAGACAACCTGGCTGAATTCAGCAAGAAATAA

The amino acid sequence of $FGBP_{1mM}$

MADTRIGVTIYKYDDNFMSVVRKAIEQDAKAAPDVQLLMNDSQNDQSKQNDQIDVLLAKGVKALAINLVDP
AAAGTVIEKARGQNVPVVFFNKEPSRKALDSYDKAYYVGTDSKESGIIQGDLIAKHWAANQGWDLNKDGQIQ
FVLLKGEPGHPDAEARTTYVIKELNDKGIKTEQLQLDTAMWDTAQAKDKMDAWLSGPNANKIEVVIANNDA
MAMGAVEALKAHNKSSIPVFGVDASPEALALVKSGALAGTVLNDANNQAKATFDLAKNLADGKGAADGTN
WKIDNKVVRVPDNVYIMADKQKNGIKAYFKIRHNVEDGSVQLADHYQQNTPIGDGPVLLPDNHYLSFQSVLS
KDPNEKRDHMVLLEFVTAAGITLGMDELYNVDGGSGGTGSKGEELFTGVVPILVELDGDVNGHKFSVSGEGE
GDATYGKLTLKLICTTGKLPVPWPTLVTTLGYGLKCFARYPDHMKQHDFFKSAMPEGYVQERTIFFKDDGNYK
TRAEVKFEGDTLVNRIELKGIGFKEDGNILGHKYVGVDKDNLAEFSKK
